# The Nociceptin/Orphanin FQ peptide receptor antagonist, SB-612111, improves cerebral blood flow in a rat model of traumatic brain injury

**DOI:** 10.3389/fphar.2023.1272969

**Published:** 2023-10-18

**Authors:** Omar N. Al Yacoub, Stefano Tarantini, Yong Zhang, Anna Csiszar, Kelly M. Standifer

**Affiliations:** ^1^ Department of Pharmaceutical Sciences, College of Pharmacy, University of Oklahoma Health Sciences Center, Oklahoma City, OK, United States; ^2^ Department of Neurosurgery, College of Medicine, University of Oklahoma Health Sciences Center, Oklahoma City, OK, United States; ^3^ Oklahoma Center for Geroscience and Healthy Brain Aging, University of Oklahoma Health Sciences Center, Oklahoma City, OK, United States; ^4^ Stephenson Cancer Center, Oklahoma City, OK, United States

**Keywords:** mild traumatic brain injury, Nociceptin/orphanin FQ (N/OFQ), cofilin-1, cerebral blood flow, Mitogen-activated protein kinases, controlled cortical impact, laser speckle contrast imaging, N/OFQ peptide receptor (NOP)

## Abstract

Traumatic brain injury (TBI) affects more than 2.5 million people in the U.S. each year and is the leading cause of death and disability in children and adults ages 1 to 44. Approximately 90% of TBI cases are classified as mild but may still lead to acute detrimental effects such as impaired cerebral blood flow (CBF) that result in prolonged impacts on brain function and quality of life in up to 15% of patients. We previously reported that nociceptin/orphanin FQ (N/OFQ) peptide (NOP) receptor antagonism reversed mild blast TBI-induced vestibulomotor deficits and prevented hypoxia. To explore mechanisms by which the NOP receptor-N/OFQ pathway modulates hypoxia and other TBI sequelae, the ability of the NOP antagonist, SB-612111 (SB), to reverse TBI-induced CBF and associated injury marker changes were tested in this study. Male Wistar rats randomly received sham craniotomy or craniotomy + TBI via controlled cortical impact. Injury severity was assessed after 1 h (modified neurological severity score (mNSS). Changes in CBF were assessed 2 h post-injury above the exposed cortex using laser speckle contrast imaging in response to the direct application of increasing concentrations of vehicle or SB (1, 10, and 100 µM) to the brain surface. TBI increased mNSS scores compared to baseline and confirmed mild TBI (mTBI) severity. CBF was significantly impaired on the ipsilateral side of the brain following mTBI, compared to contralateral side and to sham rats. SB dose-dependently improved CBF on the ipsilateral side after mTBI compared to SB effects on the respective ipsilateral side of sham rats but had no effect on contralateral CBF or in uninjured rats. N/OFQ levels increased in the cerebral spinal fluid (CSF) following mTBI, which correlated with the percent decrease in ipsilateral CBF. TBI also activated ERK and cofilin within 3 h post-TBI; ERK activation correlated with increased CSF N/OFQ. In conclusion, this study reveals a significant contribution of the N/OFQ-NOP receptor system to TBI-induced dysregulation of cerebral vasculature and suggests that the NOP receptor should be considered as a potential therapeutic target for TBI.

## Introduction

TBI is caused by an external force that causes alteration in brain physiology or pathology (Menon et al., 2010). TBI affects more than 50 million people each year (Maas et al., 2017), yet the FDA has not approved any therapeutic agents to treat TBI consequences (Vanderploeg et al., 2005; Dean and Sterr, 2013). The complicated pathophysiological consequences of TBI lead to significant disruption of delivery and increased consumption of oxygen in the brain which often results in cerebral ischemia (Algattas and Huang, 2013). According to the Mild TBI Committee of the American Congress of Rehabilitation Medicine (ACRM), mTBI severity is identified based on the Glasgow Coma Scale score of 13–15 and the severity and number of symptoms suffered by patients (Lefevre-Dognin et al., 2021). The primary tissue damage following TBI, and the subsequent events, impair the physiologic control of cerebral circulation and lead to cerebral vasospasms (Lewelt et al., 1980; Algattas and Huang, 2013), and disruption of the blood brain barrier (BBB) that contributes to vasogenic edema. Other TBI consequences that also contribute to impaired CBF include cerebral ischemia, cytotoxic edema, and increased intracranial pressure (ICP) (Lewelt et al., 1980; Algattas and Huang, 2013; Hinson et al., 2015). Both clinical and preclinical studies demonstrated that secondary pathophysiological consequences of TBI including anxiety, hyperthermia, and seizures may increase metabolic demand following brain injury (Awwad et al., 2015; Hinson et al., 2015; Stocchetti et al., 2015). Continued hemodynamic dysregulation following TBI may produce further apoptosis and necrosis in affected brain tissue. However, acute interventions to restore decreased CBF following TBI may protect brain tissue from further damage (Salehi et al., 2017).

The FDA approved the use of TBI blood biomarkers, glial fibrillary acidic protein (GFAP), and ubiquitin C-terminal hydrolase (UCH-L1), to evaluate the utility of imaging tests in adult mTBI patients (FDA, 2018). Other biochemical changes related to TBI pathology include upregulation of the axonal damage protein, neurofilament light chain (NF-L) (Liliang et al., 2010; Pandey et al., 2017; Kochanek et al., 2018; Iverson et al., 2022; Castaño-Leon et al., 2023), and upregulation and activation of the cytoskeleton-associated protein, Cofilin-1 (Campbell et al., 2012; Bahader et al., 2023) that is involved in actin filament dynamics and depolymerization.

Several animal models have been utilized experimentally to examine the biomechanical aspects of brain injury and to understand the detrimental, complex molecular cascades that are initiated by head trauma. The controlled cortical impact injury (CCI) is a mechanical model of TBI that uses a pneumatic or electromagnetic impact device to drive a rigid impactor onto the surgically exposed intact cortical dura. The CCI model produces morphologic and cerebrovascular injury responses that mirror aspects of human focal TBI. CCI impairs cerebral hemodynamic autoregulation relative to the severity of the impact and causes acute and prolonged reductions in CBF in the pericontusional cortex (Kroppenstedt et al., 2000; Liu et al., 2002; Zhang et al., 2002; Cherian and Robertson, 2003; Kroppenstedt et al., 2003; Cherian et al., 2004; Cherian et al., 2007; Liu et al., 2018).

The NOP receptor, the fourth member of the opioid receptor superfamily (Bunzow et al., 1994; Chen et al., 1994; Fukuda et al., 1994; Mollereau et al., 1994; Wang et al., 1994; Wick et al., 1994; Pan et al., 1995), and its endogenous neuropeptide, N/OFQ, are expressed in astrocytes, microglia, and neurons in the central and peripheral nervous and immune systems (Mollereau et al., 1996; Al Yacoub et al., 2022). Numerous studies demonstrated that N/OFQ levels in CSF (Armstead, 2000b; c) and brain tissue increase following injury (Witta et al., 2003; Awwad et al., 2018). These changes start early and last for a few days after the injury. Armstead’s group was the first to establish a link between N/OFQ levels upregulation and vasoconstriction of cortical cerebral arteries following cerebral ischemia, hypoxia, and TBI (Armstead, 2002; Al Yacoub et al., 2022). Administration of N/OFQ onto the exposed healthy cortex of newborn piglets induced pial artery dilation (Armstead, 1999), However, this process was reversed post-TBI. Topical N/OFQ to the injured cortex following TBI produced vasoconstriction (Armstead, 2000c), and pre-administration of a single dose of NOP receptor partial agonist attenuated the impaired cerebral vasoconstriction caused by TBI (Armstead, 2000c). We previously reported hypoxia in rat brains 8 days post-blast TBI, that was prevented by a single dose of the NOP antagonist, SB-612111 injected 30 min following blast (Awwad et al., 2018). Armstead’s work also indicated that several Mitogen-activated protein kinases (MAPKs) were involved in N/OFQ-induced vasoconstrictive actions following TBI. The studies herein investigated the effect of acute NOP receptor antagonist administration on CBF following mTBI in rats, and the effect of TBI on the N/OFQ-NOP receptor system and injury markers, cofilin-1, and MAPKs.

## Methods

### Animals

Male Wistar Han wildtype rats (*N* = 14) were purchased from Charles River Labs (Wilmington, MA) and allowed to acclimate for at least 7 days after arrival. Rats (200–300 g; 12–14 weeks of age) were housed in the animal facility under a 12-h light:12-h dark cycle (lights on at 0600) with free access to food and water. Experimental protocols were approved by the institutional animal care and use committee (IACUC) of the University of Oklahoma Health Sciences Center (OUHSC), and studies were conducted in compliance with animal welfare act (AWA) regulations, Animal Research: Reporting of *In Vivo* Experiments (ARRIVE) guidelines 2.0 (Percie du Sert et al., 2020), and other federal statutes relating to animals and experiments involving animals. Rats were randomly assigned to receive either sham or mTBI surgery.

### Controlled cortical impact (CCI)

**FIGURE 1:**
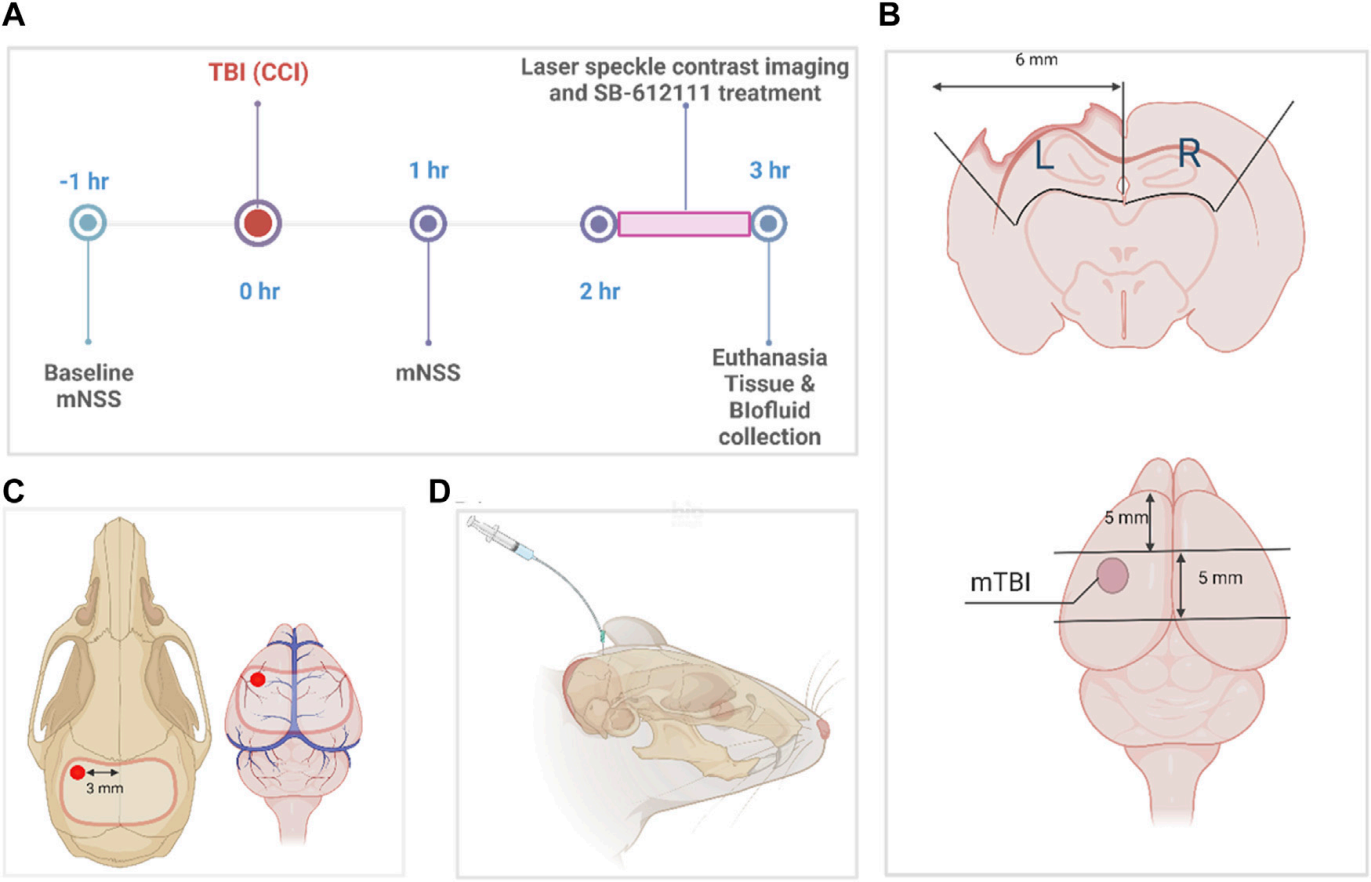
Experimental timeline and protocol diagrams. **(A)** indicates the times different data points and surgeries were performed. **(B)** illustrates tissue sample dissection of sham and mTBI brains for biochemical assays, while the size and location of the craniotomy performed for sham and mTBI surgeries and the location of the impact for mTBI is shown in **(C)**. **(D)** illustrates CSF collection from the direct insertion of a 26-gauge needle into the cisterna magna **(D)**. The dissection protocol in **(B)** was employed to combine tissue from parts of the somatosensory and motor cortex, corpus callosum, and hippocampus immediately below the area of impact (pericontusional area) and the comparable area in sham animals, to compare biochemical changes in tissues from the two groups. It observes anatomical borders of brain regions and specified dimensions to collect tissue from the ipsilateral and contralateral sides of the injury. This figure was created in BioRender.com.

CCI was performed as previously described (Brody et al., 2007; Osier et al., 2015; Osier and Dixon, 2016) with modifications to enable CBF measurements on the uninjured contralateral side at the same time as the ipsilateral side as illustrated in Figure 1. Anesthetized rats (4% isoflurane with medical air induction; 2.5%–3% maintenance) underwent stereotaxic surgery with a midline incision, exposure of the skull using a retractor, and assignment of bregma as a reference using the stereotaxic manipulator (Stoelting Co., Wood Dale, IL). Control (sham) injury animals received a 9–11 mm craniotomy (Figure 1C) that spanned from the left parietal cortex to the right parietal cortex using a hand-held drill without impact, and durotomy to expose the brain cortex.

TBI rats received a craniotomy followed by a mild controlled cortical impact (Figure 1C) with stereotaxic coordinates (1.8 mm posterior, 3.0 mm lateral to the left of the bregma) using the Impact One device (Leica Biosystems, IL) and the following actuator settings: Impactor flat tip diameter (2 mm), velocity (3 m/s), dwell time (100 m) and impact depth (4 mm). Because the animals needed to be transported to a different building to assess CBF, the bone flap was sealed in place with sterile bone wax, wounds were sutured with staples and tissue adhesive, and topical antibiotic ointment applied after each surgery. Rats remained under anesthesia sham or TBI impact until the craniotomy wound was sutured. Righting reflex time was recorded for each rat and defined as the time it took to stand on all 4 paws once anesthesia was discontinued. Body temperature and vital functions were monitored throughout surgery. Temperature was monitored using a rectal probe connected to a monitor and heating pad that adjusted temperature based on rat’s body temperature. Respiration was monitored by changes in breathing pattern (e.g., gasping, labored breathing) and/or cyanotic ears, tail, or feet. If present, isoflurane was reduced to increase oxygen delivery. Previous studies reported no changes in mean arterial blood pressure or arterial blood gasses at 30 min to 48 h following mild CCI in rats (Bryan et al., 1995; Smith and Hall, 1996; Kroppenstedt et al., 1999; Thomale et al., 2002a; Thomale et al., 2002b; Thomale et al., 2006; Muller et al., 2021).

### Modified neurological severity score (mNSS)

The mNSS (Chen et al., 2001) was used to validate the severity of the injury as a measure of overall neurological function at baseline and at 1 h following surgery. The evaluation indices included a battery of motor (raising rat by the tail (0–3); walking on floor (0–3)), sensory (proprioceptive test (0–1); visual and tactile test (0–1)), Reflex: Pinna reflex (0–1); Corneal reflex (0–1); Startle reflex (0–1), resting movement (seizures, myoclonus, myodystony (0–1)), and beam balance (0–6) tests, where normal function received a value of 0. Neurological deficit severity was categorized based on cumulative score: Severe = 13–18, moderate = 6–12, mild = 1–6 (Chen et al., 2001). Rats lacking neurological deficits scored less than 1.

### Laser speckle contrast imaging to measure CBF responses

Laser Speckle Contrast Imaging (LSCI) technology was used to measure CBF in rats following TBI. Rats were placed in the isoflurane induction chamber for 5 min (isoflurane 4%), and anesthesia was then maintained with isoflurane (2%–3%) through the nose mask while the rats were in the stereotaxic frame. The temperature was controlled using a homeothermic controller. Once rats were in the stereotaxic frame and deep anesthesia was confirmed (no toe pinch or eye blinking reflexes), the incision was opened using a sterile blade on a scalpel. Bone wax and the skull bone flap from the craniotomy were removed to expose the cranial window for LSCI. The LSCI device (Perimed, Järfälla, Sweden) was positioned above the cranial window surface (the exposed dura mater). Drops of sterile saline were applied periodically over the exposed dura mater to keep it and cortical tissue moist during imaging. After initial CBF readings were obtained, the direct effect of vehicle (5% dimethylsulfoxide and 0.9% sodium chloride) on CBF was assessed before and after the topical application of sterile SB-612111 onto the exposed cortex with sterile pipettes for 5–10 min or until each measurement returned to baseline. To evaluate changes in CBF, an elliptic shape, with dimensions of ∼4 mm (height) and 2 mm (width), was drawn over each side of the brain in live images of each rat using the PIMsoft software (Perimed, Järfälla, Sweden). The inner borders of the shapes over each side were ∼1 mm from the midline between the two hemispheres. To identify reductions in CBF on the ipsilateral side after TBI or sham surgery, the relative percent change of CBF in the ipsilateral side to the contralateral side was generated by the software. To identify changes in CBF following the application of sequential doses of SB-612111, times of interest (TOI) in the CBF graph of each side were identified after each dose, and percent change relative to the baseline at each TOI after injury was generated and used for statistical analysis. Three rats were excluded: one died during anesthesia; excessive bleeding prevented CBF assessment for two others. The total number of rats with CBF assessments per group was 5 sham and 6 mTBI.

### NOP antagonist (SB-612111) preparation and treatment

[(−)-cis-1-Methyl-7-[[4-(2,6-dichlorophenyl) piperidin-1-yl] methyl]-6,7,8,9-tetrahydro-5H-benzocyclohepten-5-ol], SB-612111 (SB; Tocris Bioscience, Bristol, United Kingdom) was dissolved in sterile 5% dimethylsulfoxide and 0.9% sodium chloride vehicle to improve solubility and absorption of the drug. Three final concentrations (1 μM, 10 μM, and 100 µM) of the SB were applied sequentially to the exposed cortex following assessment with vehicle 2 h following CCI. SB concentrations were selected based on results from previous studies (Spagnolo et al., 2007; Liao et al., 2011; Vazquez-DeRose et al., 2013).

### Processing and collection of biofluid and brain tissue samples

Rats were euthanized via intracardiac exsanguination under isoflurane anesthesia, wherein brain, CSF, and blood were collected. After whole blood cardiac exsanguination, whole blood was stored at room temperature for 30 min, the supernatant (serum) was collected after centrifugation at 5,000 × *g*, 4°C for 5 min (Shear et al., 2016) and flash frozen in 250 µL aliquots. CSF (∼100–200 µL) was collected from the direct insertion of a 26-gauge needle into the cisterna magna (Figure 1D). Brains were extracted and dissected using a matrix brain slicer (Zivic Instruments) to include separate 5 mm sections of ipsilateral (left) and contralateral (right) tissue (cortex, corpus callosum, and hippocampus) as illustrated in Figure 1B. Brain tissue was then homogenized and divided for radioimmunoassay, qPCR, and immunoblotting then were processed and stored in −80°C as described previously (Al Yacoub et al., 2023). We considered the general effect of topical SB on the dissected tissue to be negligible since it was present for only a short time and was washed away such that CBF returned to pre-SB treatment levels before rats were euthanized.

### Radioimmunoassay (RIA)

Peptide extraction to assess N/OFQ content from tissue homogenates, CSF and serum was performed in duplicate samples using an RIA kit (Phoenix Pharmaceuticals, Belmont, CA) as described in the manufacturer’s protocol. The concentration of soluble protein present in the brain tissue extract was determined by the BCA method. The total amount of N/OFQ immunoreactivity (IR) was calculated and expressed as pg/mL in CSF and serum samples, and as pg/mg for tissue samples. Samples were excluded if they fell outside of the range of the standard curve or if contaminated with blood.

### Immunoblotting

Frozen tissue homogenates were thawed and treated with cell lysis buffer (50 mM Tris pH 7.5, 0.5 M NaCl, 50 mM NaF, 10 mM EDTA, 2 mM EGTA, 1% Triton X-100, 2 mM Na_3_VO_4_, 10 µM Na_4_P_2_O_7,_ 250 µM PMSF) with freshly added protease and phosphatase inhibitor cocktail (Santa Cruz Biotechnology, Dallas, TX). The protein concentration in supernatants (14,000 x g at 4°C for 20 min) was measured using a BCA protein assay kit (Pierce™, ThermoFisher Scientific Inc.), then samples were solubilized in 4X sample loading buffer (LI-COR Biosciences, Lincoln, NE) and heated to 65°C for 20 min. Samples (20 µg of total protein) were resolved by Novex™ WedgeWell™ 8%–16% gradient Tris-Glycine gels (Thermo Fisher Scientific Inc.), transferred to nitrocellulose membranes and probed for the following proteins: GFAP (GPCA-GFAP, 1:4000; EnCor Biotechnology, Gainesville, FL), UCH-L1 (sc-271639, 1:200; Santa Cruz Biotechnology), NF-L (sc-20012, 1:200; Santa Cruz Biotechnology), and actin (A3853, 1:2000; Sigma-Aldrich). MAPK antibodies were purchased from Cell Signaling Technology, Beverly, MA, and were diluted as follows: ERK1/2 (4696S, 1:2000), phospho-ERK1/2 (4370S, 1:500), p-38 MAPK (9228S, 1:1000), SAPK/JNK (9252S, 1:1000). Blots were incubated in primary antibody overnight at 4°C and secondary antibody for 1 h at room temperature. IRDye^®^ 800CW goat anti-rabbit (1:10000), IRDye^®^ 680CW donkey anti-rabbit (1:10000), IRDye^®^ 680CW donkey anti-mouse (1:10000), IRDye^®^ 800CW donkey anti-mouse (1:10000), IRDye^®^ 800CW donkey anti-goat (1:10000), IRDye^®^ 680CW goat anti-mouse (1:10000), were purchased from LI-COR Biosciences (Lincoln, NE). Blots were processed, images captured, and band density analyzed using the Odyssey^®^ CLx Infrared Imaging System (LI-COR Biosciences, Lincoln, NE). Band density was normalized to the loading control actin in the corresponding lane using Image Studio™ Lite image processing software Ver 5.2 (LI-COR Biosciences, Lincoln, NE). Quantification of the GFAP bands included the GFAP breakdown product bands.

### Real-time quantitative PCR (qPCR)

TriPure reagent (Sigma-Aldrich, MO) was immediately added to brain tissue homogenate collected for mRNA extraction and stored at −80°C. cDNA was synthesized using Super-Script III Reverse Transcriptase (Sigma-Aldrich, MO). Real-time PCR was performed using PowerUp™ SYBR™ Green Master Mix (Applied Biosystems, Foster City, CA) and 125 nM forward and reverse primers of target genes (rat GAPDH Fwd: 5′-ACC CAG AAG ACT GTG GAT GG-3′, Rev: 5′-CAC ATT GGG GGT AGG AAC AC-3′; rat NOP Fwd: 5′-GTT CAA GGA CTG GGT GTT CAG CCA GGT AGT-3′; rat NOP Rev: 5′-TGC TGG CCG TGG TAC TGT CTC AGA ACT CTT-3′; rat preproN/OFQ Fwd: 5′-TGC ACC AGA ATG GTA ATG TG-3′, Rev: 5′-TAG CAA CAG GAT TGT GGT GA-3′, all from Sigma-Aldrich) in an ABI 7000 Sequence Detection System (Applied Biosystems, CA). GAPDH gene was used as an internal standard to which expression of other genes was normalized. Data were analyzed using the comparative *C*t method and compared to control values from sham rats (Schmittgen and Livak, 2008).

### Data Analysis

Data Analysis and graph preparation were performed using GraphPad Prism 9.5.1 software (GraphPad Software, La Jolla, CA, United States). Data are expressed as mean ± SD unless indicated otherwise. Statistical comparisons were performed by two-way ANOVA with Tukey’s post-hoc analyses as automatically recommended by the software, or a two-tailed, unpaired student’s t-test as appropriate. Results were considered statistically significant if *p* < 0.05. All data were subjected to Shapiro-Wilk (N < 8) normality tests before analysis. Those groups that failed the normality test (*p* < 0.05) were subjected to an outlier test (ROUT; Q = 1%) as recommended, to determine if the outlier was responsible for the failed normality test. Pearson’s Correlation Analysis was performed with the following data aligned from each rat: tissue N/OFQ in the ipsilateral side of the brain and in CSF, differences in CBF in the ipsilateral side relative to contralateral after injury, and injury markers. Correlations were made with data from sham and mTBI groups.

## Results

**FIGURE 2:**
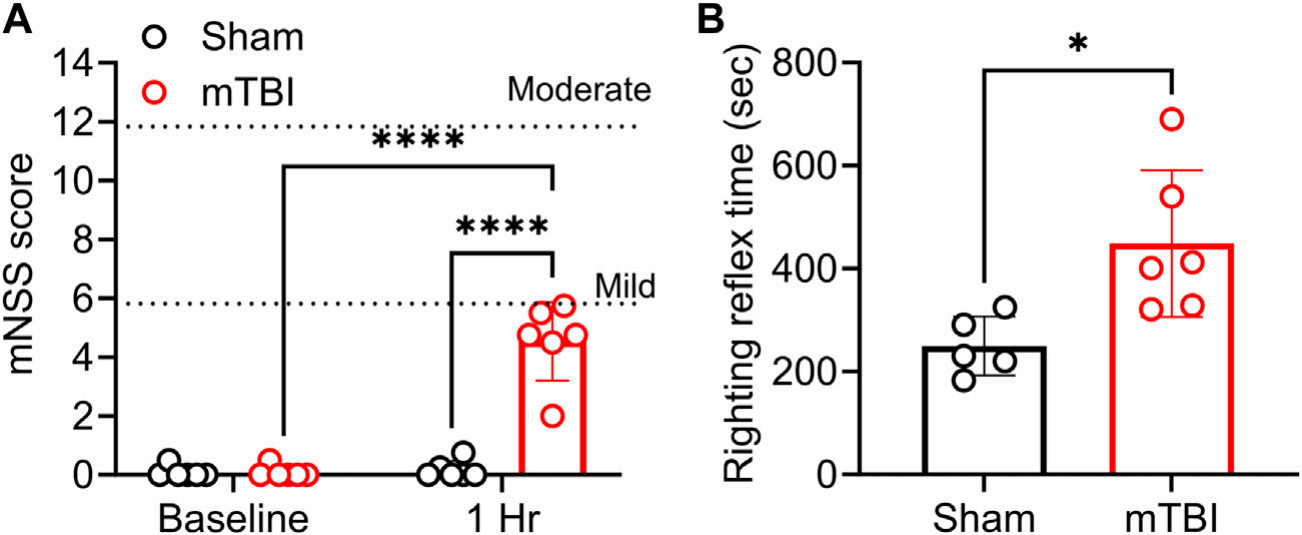
CCI produced mild TBI and prolonged the righting reflex time. TBI severity was determined by mNSS assessment; mNSS scores 1 h post-TBI and sham surgery are shown in **(A)**; righting reflex time is found in **(B)**. Both are presented as a scatter plot with mean ± SD (*n =* 5-6 per group). Dotted lines in panel **(A)** at 6 and 12 represent the upper limit of mild and moderate severity, respectively. Severe injury scores range from 13–18. Significant differences are represented as *p < 0.05 and ****p < 0.0001. Analysis of **(A)** was performed with two-way ANOVA with Tukey’s multiple comparisons tests, while results in **(B)** were analyzed using a student’s two-tailed unpaired *t*-test.

CCI TBI produces mild severity injury with prolonged righting reflex time 1 h post-impact. To assess overall neurological function and to validate the severity of the impact produced, mNSS scores were determined before surgery (baseline) and at 1 hour following sham or TBI injury. No rats were excluded prior to TBI as all scored less than 1 (in the normal range). Two-way ANOVA analysis showed a significant interaction between injury and time [F (1, 20) = 58.58, *p*= <0.0001], the effect of injury [F (1, 20) = 58.58, *p*= <0.0001], and time [F (1, 20) = 63.13, *p*= <0.0001]. All rats that received mTBI yielded mNSS scores within the mild severity range (mNSS = 1–6) 1-h following injury (Figure 2A). TBI also prolonged righting reflex time compared to sham rats (*p* = 0.017; Figure 2B).

**FIGURE 3:**
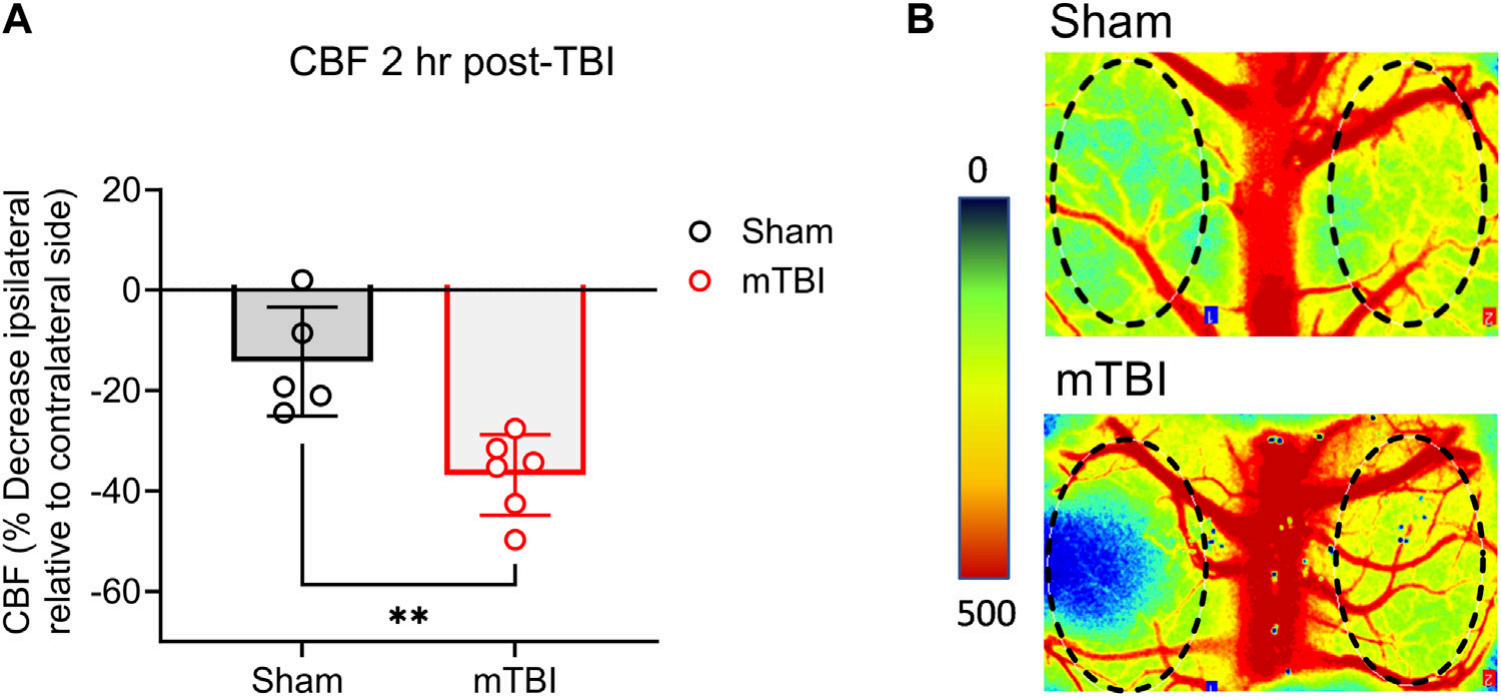
mTBI reduces CBF. **(A)** Values are presented as a scatter plot including mean ± SD (*n* = 5-6 per group). **(B)** Perfusion maps of the rat brain are visualized using LSCI and were pseudocolored using an arbitrary color map. Significant differences are represented as **p < 0.01 according to analysis with two-tailed, unpaired student’s t-test.

mTBI reduces CBF. Baseline CBF assessments could not be made due to instrumental limitations inherent to LSCI and the fact that the impactor and LSCI devices were located in two different buildings. Therefore, to assess the effect of mTBI on CBF, a reduction in CBF on the ipsilateral side was calculated in sham and CCI surgery animals relative to the same animal’s contralateral side using the PIMsoft software as described in the methods section. TBI reduced CBF to a greater extent 2 h post-surgery than rats that received sham surgery (*p* = 0.0032; Figure 3A). Representative CBF images of ipsilateral and contralateral sides of sham and CCI surgery rats are shown in Figure 3B. Areas of highest blood flow appear as bright red, while dark blue indicates the lowest levels of blood flow.

**FIGURE 4:**
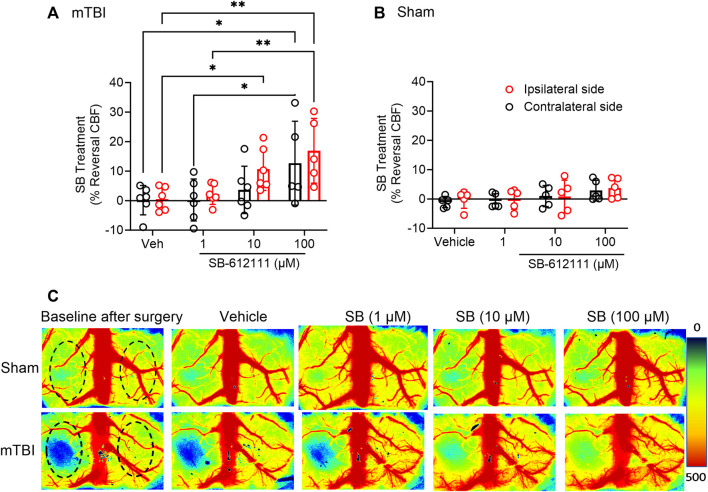
Topical application of SB-612111 improved CBF in the ipsilateral side following mTBI, but not sham surgery. SB improved CBF in the ipsilateral side following mTBI after addition of 10 µM and 100 μM, dropwise **(A)**. Increased CBF on contralateral side in mTBI rats was noted only following 100 μM SB, compared to vehicle but not the contralateral side compared to vehicle. Vehicle and SB treatment has no effect on CBF following sham **(B)**. **(C)** contains representative images of CBF in the cortex following sham (upper panels) and mTBI (lower panels) at after surgery and following treatment with vehicle, 1 μM, 10 μM, and 100 µM SB. Values are presented as mean ± SD. Significant differences are indicated as **p < 0.01; *p < 0.05 as determined by Repeated measures 2-way ANOVA with Tukey’s post-hoc test.

NOP receptor antagonist treatment improved CBF following mTBI but not after sham surgery. The vehicle and three concentrations of SB were applied topically, stepwise, to the dura once initial CBF measurements were completed. After each application, CBF was recorded until it stabilized (5–10 min) before the next application was made. CBF improved in the ipsilateral hemisphere following topical application of 10 µM (*p* = 0.0380) and 100 µM (*p* = 0.0011) SB to the exposed cortex of mTBI rats compared to vehicle application; 100 uM SB increased CBF significantly more than 1 uM SB (*p* = 0.0037; Figure 4A). CBF increased on the contralateral hemisphere in mTBI rats only with 100 µM SB compared to vehicle (*p* = 0.0146) and to 1 uM (*p* = 0.0154; Figure 4A). Two-way ANOVA analysis showed a significant effect of SB concentration on CBF [F (3, 28) = 12.31, *p*= <0.0001]. None of the SB concentrations altered CBF in either hemisphere following sham injury (Figure 4B). Representative images of CBF in sham and mTBI rats following surgery and each successive topical addition are shown in Figure 4C.

**FIGURE 5:**
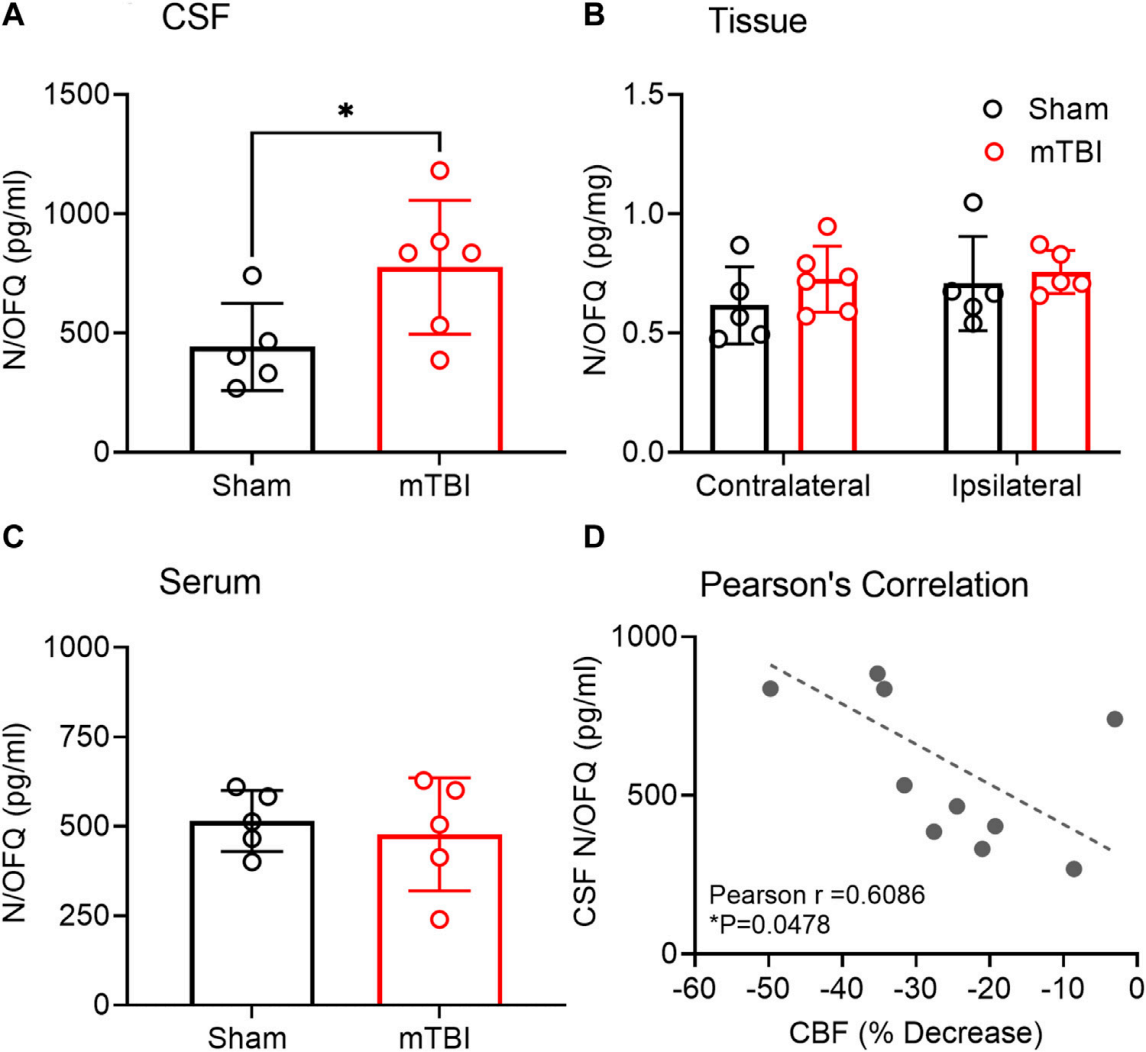
N/OFQ levels in CSF, serum, and tissue from contralateral and ipsilateral hemispheres collected 3 h post mTBI. Levels of N/OFQ were quantified using RIA in CSF **(A)** and serum **(C)** collected from rats following euthanasia 3 h post-TBI. Data were analyzed using a two-tailed unpaired *t*-test, and values are presented as mean ± SD (*n =* 5-6 per group). Differences from sham are represented as *p < 0.05). **(B)** indicates N/OFQ levels measured in ipsilateral and contralateral tissue collected 3 h post-TBI. Values are presented as mean ± SD (*n =* 5-6 per group). Two-way ANOVA with Tukey’s post-hoc test was employed to determine contributions of injury severity and side of the brain. **(D)** represents the results of a Pearson’s correlation analysis between N/OFQ levels in CSF and the % decrease in CBF on the ipsilateral side relative to the contralateral side, *p < 0.05. Two samples were excluded due to contamination with blood.

Increased N/OFQ levels in CSF following mTBI correlated with CBF decrease on the ipsilateral side. N/OFQ levels were measured in CSF, serum and tissue dissected from ipsilateral and contralateral hemispheres as illustrated in Figure 1B. Mild TBI increased N/OFQ levels 3 h post-TBI in CSF compared to sham (*p* = 0.0487; Figure 5A), but not in tissue (Figure 5B) or serum (Figure 5C). Figure 5D shows the results of the correlation analysis between percent change in CBF in the ipsilateral hemisphere relative to the contralateral hemisphere and levels of N/OFQ in CSF. N/OFQ levels in CSF negatively correlated with a percent decrease in CBF in the ipsilateral hemisphere relative to the contralateral hemisphere. No correlations between N/OFQ levels in ipsilateral tissue, or in serum and the decrease in CBF were found.

**FIGURE 6:**
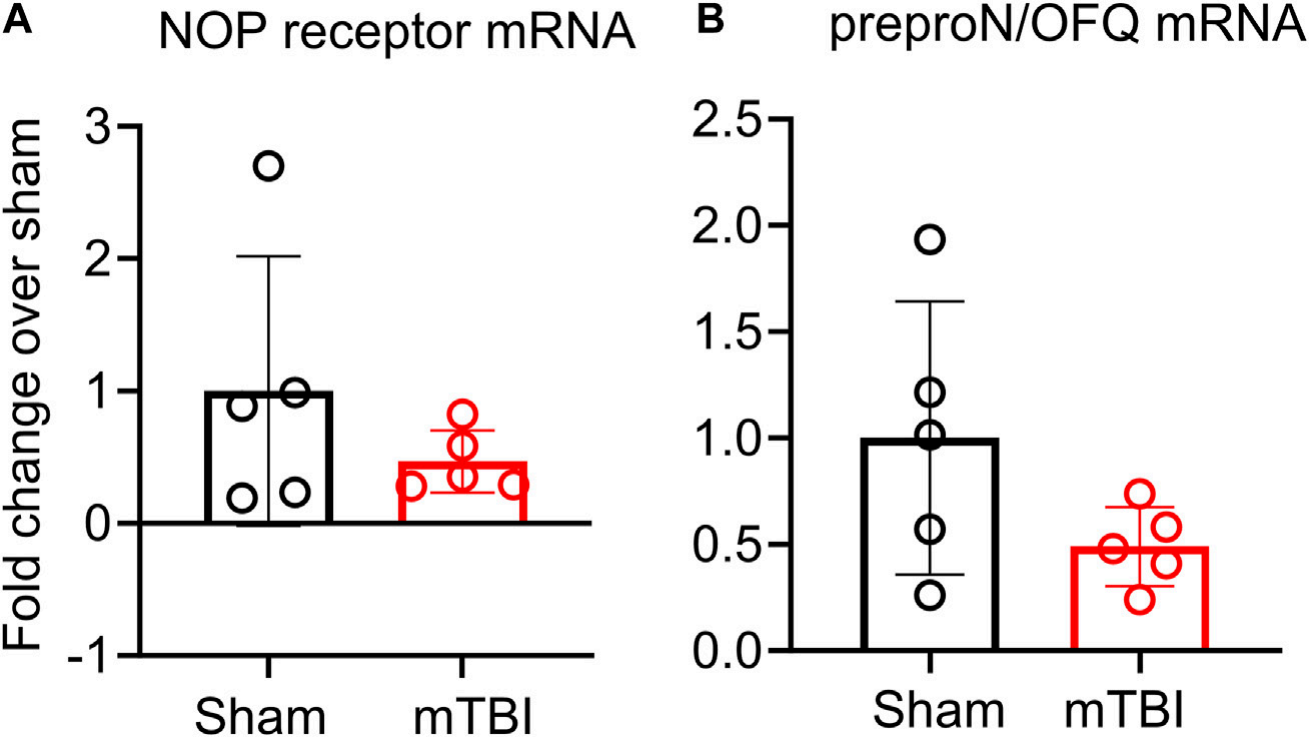
NOP receptor **(A)** and preproN/OFQ **(B)** mRNA expression in ipsilateral tissue is not altered 3 h post-mTBI. Messenger RNA was extracted from ipsilateral tissue for real-time PCR analysis as described in methods. Target gene expression in sham and mTBI-treated rat ipsilateral hemispheres were normalized to GAPDH gene expression and individual 2^−ΔΔCT^ values from mTBI (*n* = 5) were normalized to the mean of individual 2^−ΔΔCT^ values of the sham group (*n* = 6) to determine fold change in mRNA. Values are presented as mean ± SD and compared using a two-tailed unpaired student’s *t*-test.

The effect of mTBI on N/OFQ peptide and NOP receptor mRNA was also examined. Ipsilateral hemisphere tissue mRNA was prepared and subjected to real-time PCR analysis as described above. No differences in mRNA levels between sham and mTBI rats were found (Figures 6A, B).

**FIGURE 7:**
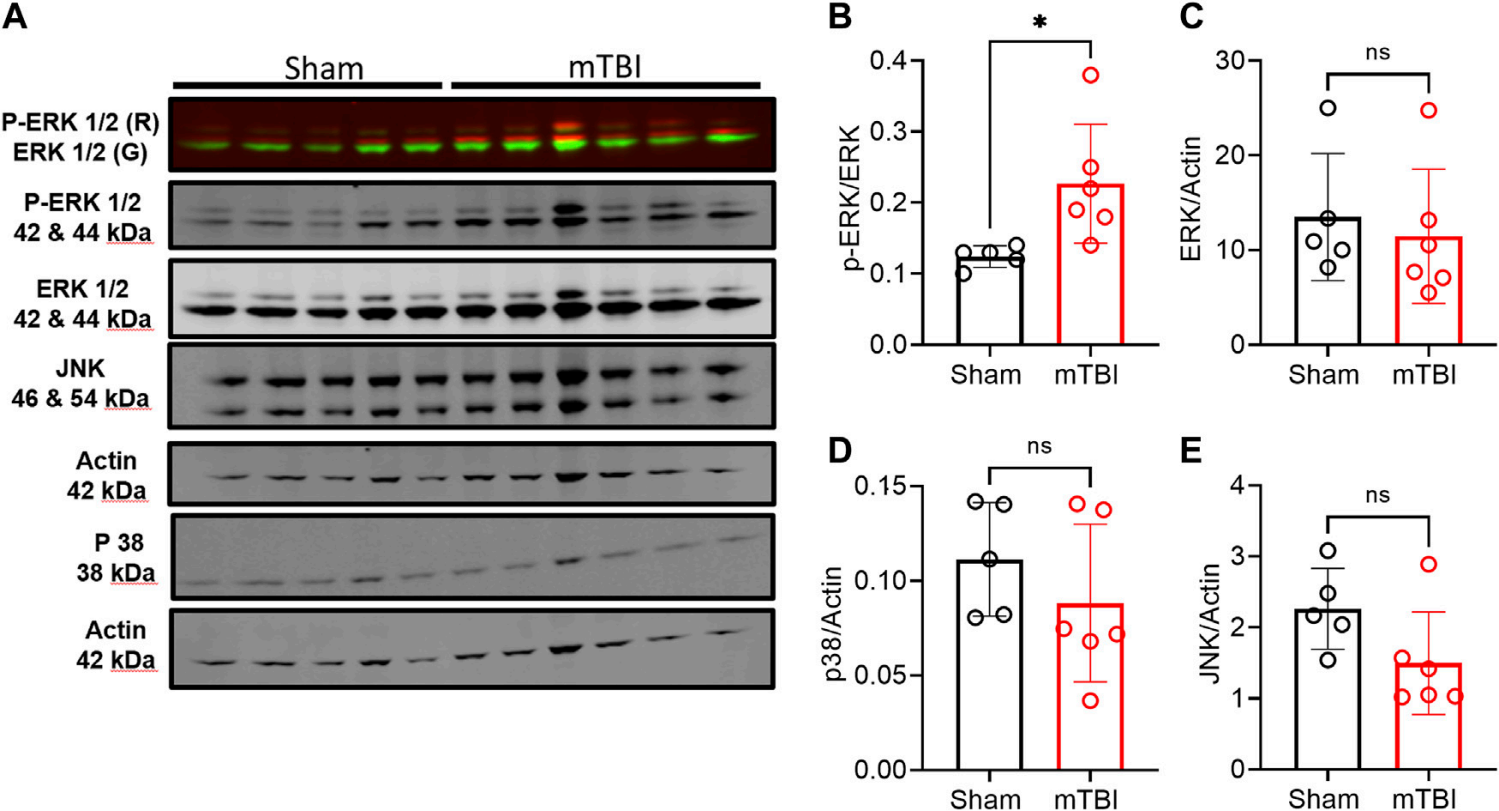
Expression of MAPKs in ipsilateral tissue from rat brains collected 3 h post-surgery. Representative immunoblots of MAPKs from brain tissue of 5–6 rats in each group at 3 h post-TBI are shown in **(A)**. Expression of p-ERK **(B)**, ERK **(C)**, p38 **(D)**, and JNK **(E)** were quantified by densitometric analysis of immunoblots normalized to actin loading control from the same lane except phospho-ERK was normalized to total ERK values. A two-tailed unpaired *t*-test was performed to assess difference from sham; significant differences are denoted by *p < 0.05. Values are presented as mean ± SD (*n =* 5-6 per group).

**FIGURE 8:**
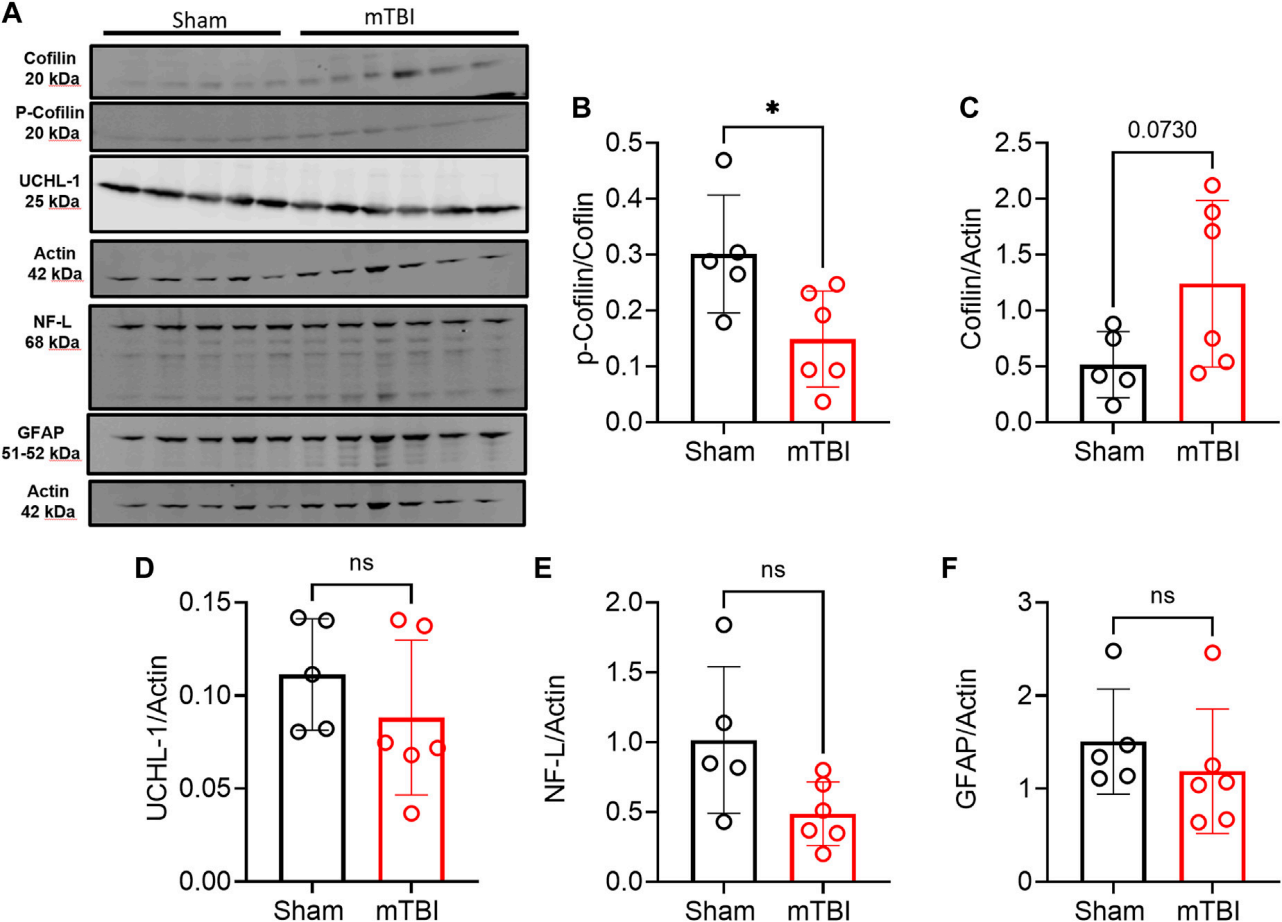
Expression of injury markers in ipsilateral tissue from rat brain hemispheres collected 3 h post-surgery. Representative immunoblots of TBI or ischemia-related markers from 5–6 rats in each group are shown in **(A)**. **(B, C)** show expression of phospho-cofilin-1 (actin depolymerizing factor) and cofilin-1 (**B, C**, respectively). Phospho-cofilin-1 was normalized to total cofilin-1 values. UCHL-1 (neuronal injury marker) **(D)**, NF-L [axonal injury marker; **(E)]**, and GFAP [astrogliosis marker; **(F)**] were quantified by densitometric analysis of immunoblots and values were normalized to actin loading control from the same lane. A two-tailed unpaired *t*-test was performed to assess the difference between TBI, and sham, and significant differences are denoted by *p < 0.05. Values are presented as mean ± SD.

mTBI increased ERK and cofilin-1 activation in ipsilateral brain tissue compared to sham. Phospho-ERK expression increased following mTBI in tissue from the ipsilateral hemisphere compared to ipsilateral tissue in sham (*p* = 0.0253; Figures 7A,B). Pearson correlation analysis between ipsilateral N/OFQ and phospho-ERK expression was performed and ipsilateral N/OFQ levels positively correlated with phospho-ERK expression (r = 0.8115, *p* = 0.0024). No differences between mTBI and sham groups in expression of total ERK, p38, and JNK in ipsilateral brain tissue 3 h post-mTBI were noted (Figures 7C–E respectively). Unlike ERK, which is activated by phosphorylation, cofilin-1 is activated by dephosphorylation (Meberg et al., 1998; Wang et al., 2005). Phospho-cofilin-1 expression decreased in ipsilateral TBI tissue compared to sham (*p* = 0.0270; Figures 8A,B), as determined by two-tailed, unpaired Student’s t-test, consistent with the presence of ischemia. No significant differences between mTBI and sham groups in expression of cofilin-1, UCH-L1, NF-L, and GFAP in ipsilateral brain tissue at 3 h post-mTBI were found (Figures 8C–F, respectively).

## Discussion

This study generated several important and novel findings to advance our understanding of the role of the N/OFQ-NOP receptor system in cerebrovascular dysregulation following focal mTBI with CCI. First, topical application of a NOP receptor antagonist onto the exposed cortex of rats within 1–2 h following mild CCI TBI improved CBF. Second, N/OFQ levels in CSF increased acutely (within 3 h of impact) following mTBI. Third, mTBI CCI increased activation of ERK MAPK and cofilin-1 within 3 h post-impact.

It is well established that CCI impairs cerebral hemodynamic autoregulation relative to the severity of the impact and causes acute and severe reductions in CBF in the pericontusional cortex (Kroppenstedt et al., 2000; Liu et al., 2002; Zhang et al., 2002; Cherian and Robertson, 2003; Kroppenstedt et al., 2003; Cherian et al., 2004; Cherian et al., 2007). The precise mechanism(s) underlying disruptions in the neurovascular unit and vasoconstriction that occur within hours following TBI remain poorly understood. However, previous findings hypothesized that vasospasm, including vasoconstriction of large and small cerebral vessels, is induced by increased blood pressure and subarachnoid hemorrhage after TBI (Izzy and Muehlschlegel, 2014), accompanied by increased transportation of endothelin receptors to the cellular membrane in the neurovascular unit (Kallakuri et al., 2007), and pericyte migration from the vascular wall (Dore-Duffy et al., 2000). Work by Armstead’s lab suggests that the N/OFQ-NOP receptor system may mediate this process by several potential mechanisms, including activation of ERK (Armstead, 2003; 2006). This is the first time that CCI-induced changes in CBF were shown to be correlated with N/OFQ levels and modulated by a NOP receptor antagonist. CCI causes acute reductions in CBF in pericontusional cortex (Kroppenstedt et al., 2000; Liu et al., 2002; Zhang et al., 2002; Cherian and Robertson, 2003; Kroppenstedt et al., 2003; Cherian et al., 2004; Cherian et al., 2007). A recent study in mice reported both acute and prolonged reductions (from 6 h to 21 days post-TBI) in cortical CBF following CCI using the laser speckle imaging approach (Liu et al., 2018). Our findings (Figure 3) support those previous reports of acute reduction in cortical CBF following CCI compared to sham. We were not able to measure cortical CBF baseline prior to sham or CCI because the LSCI apparatus was in a different building from where the surgery was performed. Therefore, the contralateral hemisphere was used as a baseline reference to evaluate the effect of mild impact or sham surgery on cortical CBF post-surgery as described in the methods section.

Based on previous findings from our group and others (Armstead, 2000c; Witta et al., 2003; Awwad et al., 2018), we hypothesized that mTBI would acutely increase N/OFQ levels in brain tissue and CSF. Our findings support the acute increase in N/OFQ levels in CSF shortly after TBI, as was demonstrated previously using the fluid percussion injury model of TBI (FPI) (Armstead, 2000c; Armstead, 2002) in piglets. However, the new findings demonstrate that N/OFQ levels in tissue are not yet upregulated at 3 h following mTBI CCI (Figure 5B), as reported for 8 days post-CCI TBI (Al Yacoub et al., 2023) 1 day post blast TBI (Awwad et al., 2018) or 6 hr–24 h following cortical stab injury (Witta et al., 2003). Similarly, this study found no increases in serum N/OFQ 3 h post-CCI (Figure 5C), as we’d previously reported for plasma 24 h post-blast injury (Awwad et al., 2018). This suggests that 3 h is too early to detect changes in N/OFQ levels in tissue or in blood. Collectively, this indicates that there is an acute release of N/OFQ into the CSF, but the process of replenishing peptide stores has not yet begun at this early time point. This is confirmed by our RT-PCR results in which no difference in N/OFQ mRNA tissue levels between sham and CCI TBI rats was evident (Figure 6B). Following stab TBI, N/OFQ mRNA was not elevated in pericontusional tissue until 24 h post-injury (Witta et al., 2003). However, the fact that N/OFQ levels in CSF correlated negatively with a percent decrease in CBF in the ipsilateral cortex relative to the contralateral cortex (Figure 5D) establishes an association between increased N/OFQ levels and mTBI-induced cerebrovascular disruption.

N/OFQ vasodilates and directly relaxes blood vessels under normal, non-injury conditions (Champion et al., 2002; Brookes et al., 2007; Simonsen et al., 2008). Systemic (Hashiba et al., 2003) and central (Burmeister and Kapusta, 2007) administration of N/OFQ reduces blood pressure and causes bradycardia. The indirect vasodilative effects of N/OFQ are mediated by histamine released from immune cells in the blood that occurs after NOP receptor activation in those cells (Brookes et al., 2007). Direct relaxation occurs by inhibiting pre-junctional adrenergic neurotransmission (Simonsen et al., 2008). The vasodilatory effect of N/OFQ is not nitric oxide or prostaglandin-dependent (Champion et al., 2002). Lambert’s group showed recently that sepsis increases NOP receptor mRNA expression in human vascular endothelial cells, but not in vascular smooth muscle cells *in vitro* (Bird et al., 2022). N/OFQ administration on cortical cerebral arteries elicited vasodilation that is protein kinase C (PKC), K(ATP), and k(Ca) activation dependent under normal conditions (Armstead, 1999). However, N/OFQ application caused vasoconstriction following ischemia and brain injury (Armstead, 2000a; Armstead, 2000b; Armstead, 2000c; Armstead, 2002). Administration of the NOP receptor putative antagonist, [F/G] NOC/OFQ (1–13), attenuated pial artery vasoconstriction and impaired CBF when applied topically to the cortex shortly after FPI TBI (Armstead, 2000c; Armstead, 2002). This peptide was later classified as a NOP receptor partial agonist, not an antagonist, based upon pharmacological studies conducted by several groups (McDonald et al., 2003; Kapusta et al., 2005; McDonald and Lambert, 2010; Asth et al., 2016). SB-612111 is a standard NOP receptor antagonist and one of the most potent and selective nonpeptide antagonists for the NOP receptor (Spagnolo et al., 2007). Our group previously demonstrated that a single treatment with SB injected shortly after mild blast TBI prevented the development of hypoxia in brain tissue of rats 8 days post-TBI (Awwad et al., 2018). One of the major goals of this study was to examine the acute effect of SB treatment on CBF (1–3 h) post-CCI TBI. We hypothesized that the acute upregulation of the N/OFQ-NOP receptor system contributes to CBF-induced deficits post-CCI TBI, and that a topical application of SB to the exposed cortex would attenuate the decrease in CBF following CCI TBI. Three different SB concentrations were applied to the exposed cortex, 1–100 μM, 2 h post-TBI. Two concentrations, 10 and 100 μM, improved CBF in ipsilateral tissue following TBI. No effects on sham rat CBF were found. The lack of effect of SB on CBF in sham was likely because CSF N/OFQ levels remained unchanged from baseline in those rats. Since we did not measure baseline CBF before surgery, it is not known if, or to what extent, CBF in the contralateral side was also affected by TBI. These findings improve our understanding of one of the mechanisms by which the N/OFQ-NOP receptor system contributes to TBI-induced deficits. However, further studies are needed to explore the effect of systemic administration of NOP receptor antagonists on cerebrovascular dysregulation post-TBI.

TBI also upregulates and activates MAPKs (Zeng et al., 2023). ERK and p38 MAPKs and protein kinase C (PKC) activation were involved in N/OFQ-mediated vasoconstrictive actions in the parietal cortex post-FPI TBI (Armstead, 2003; Philip and Armstead, 2003; Ross and Armstead, 2005). In this study, we evaluated changes in MAPKs and other injury markers in pericontusional tissue collected from sham and mTBI animals. We considered the general effect of topical SB on the dissected tissue to be negligible since it was present for only a short time and was washed away until previous CBF levels returned. This appears to be a fair assessment as increased phosphorylation of ERK was detected 3 h post-TBI in ipsilateral tissue compared to sham (Figure 7A). The levels of ERK phosphorylation correlated with tissue N/OFQ levels, which indicates an association between N/OFQ levels and activation of ERK. However, no changes in phosphorylation or total protein levels of p38 and JNK were detected at this early time point. Further studies are needed to link the timeline of vasoconstrictive actions and decreased CBF post-TBI with N/OFQ-NOP receptor system-mediated activation of MAPKs.

Acute elevations (2 h–5 days) in UCH-L1 (Liu et al., 2010; Osier et al., 2021) and NF-L (Korley et al., 2018; Karlsson et al., 2021) levels were previously detected in serum and CSF post-TBI. The earliest time at which changes in GFAP levels in injured brain tissue was detected is 3 days post-TBI (Wang et al., 2018; Niu et al., 2020), and our results are consistent with that. One of our aims in this study was to evaluate the effect of mTBI on injury markers in injured tissue at 3 h post-TBI. We found no changes in levels of UCH-L1, GFAP, or NF-L in injured tissue at this early time point (Figure 8) compared to sham. This is the first study to report on tissue levels of NF-L and UCH-L1 at 3 h post mild CCI TBI.

Cofilin-1 is a cytoskeleton-associated protein involved in actin filament dynamics and depolymerization (Pollard et al., 2000). TBI and cerebral ischemia increased cofilin expression and its de-phosphorylation in injured tissue, and it has been used as a marker of ischemia (Campbell et al., 2012; Bahader et al., 2023). Activation of cofilin by de-phosphorylation (e.g., reduced phosphorylation) leads to increased actin depolymerization and, as a result, dendritic remodeling and spine loss post-TBI (Campbell et al., 2012). Cofilin-1 activation is also involved in oxidative stress and microglial activation responses post-TBI (Bahader et al., 2023). Activated cofilin may be involved in BBB disruption by destabilizing tight junction adherent junction proteins connecting endothelia cells within the BBB (Toshima et al., 2001; Suurna et al., 2006; Nagumo et al., 2008; Shiobara et al., 2013), thus it may also become a useful marker of BBB integrity. Several signaling pathways are involved in cofilin-1 phosphorylation and dephosphorylation under physiological and pathophysiological conditions in the CNS (Namme et al., 2021). However, regulation of cofilin-1 expression and activation following TBI is not well studied. Our results supported findings from the few previous studies (Campbell et al., 2012; Bahader et al., 2023) that show an early activation of cofilin by dephosphorylation in ipsilateral tissue post-TBI. This activation of cofilin is an indication of the acute ischemic response in injured tissue resulting from tissue damage and decreased CBF post-TBI. No correlation was found between cofilin-1 or p-cofilin-1 expression in tissue, and N/OFQ levels in tissue or CSF. Further studies at later timepoints are needed to better understand if there is a relationship between N/OFQ and cofilin-1 activation, as well as the mechanism, in general, behind cofilin-1 activation and upregulation post-TBI.

## Conclusion

The present study demonstrated the involvement of the N/OFQ-NOP receptor in decreased CBF 1–3 h post-mTBI. Mild TBI results in decreased CBF, ischemia, increased release of N/OFQ into the CSF, and activation of ERK MAPK as early as 3 h post-CCI TBI, while other injury markers and MAPKs were unchanged at this early time point. Together, our data suggest that acute blockade of the NOP receptor provides a protective effect against cerebrovascular dysregulation and potentially prevents the detrimental effects of decreased CBF post-mTBI.

## Scope and research topic statement

We submit our manuscript as an original research article to Frontiers in Pharmacology as part of the Neuropharmacology Research Topic: Therapies for brain injury. Our manuscript falls under the theme of ‘Preclinical Pharmacology: animal models, validation of novel drug targets, the mechanism(s) of drug action, innovative methods for drug delivery to the brain’. Our study reveals a significant contribution of the Nociceptin/Orphanin FQ (N/OFQ)- Nociceptin/Orphanin FQ peptide (NOP) receptor system to TBI-induced dysregulation of cerebral vasculature and suggests that the NOP receptor should be considered as a potential therapeutic target for TBI. We utilized one of the 3 most widely used TBI models, controlled cortical impact (CCI), to produce mild TBI, and used the NOP receptor antagonist SB-612111 (SB) to dose-dependently reverse TBI-induced reductions in CBF. N/OFQ levels increased in the cerebral spinal fluid (CSF) acutely following mTBI, which correlated with the percent decrease in ipsilateral CBF. TBI also activated ERK and cofilin within 3 h post-TBI, and ERK activation positively correlated with increased CSF N/OFQ. These findings support the findings of a recent publication that demonstrate that recovery from CCI injury in NOP receptor −/− rats is more rapid and complete than in WT.

## Data Availability

The raw data supporting the conclusion of this article will be made available by the authors, without undue reservation.

## Abbreviations

CBF, Cerebral blood flow; CSF, Cerebral spinal fluid; CCI, Controlled cortical impact; ERK, Extracellular signal regulated kinase; GFAP, Glial fibrillary acidic protein; MAPK, Mitogen-activated protein kinase; mNSS, Modified neurological severity score; mTBI, Mild traumatic brain injury; N/OFQ, Nociceptin/Orphanin FQ; NF-L, Neurofilament light chain; NOP, nociceptin/orphanin FQ (N/OFQ) peptide; PpN/OFQ, Prepronociceptin; SB, SB-612111; TBI, Traumatic brain injury; UCH-L1, Ubiquitin carboxy-terminal hydrolase—L1.
